# Engineering Heteromaterials to Control Lithium Ion Transport Pathways

**DOI:** 10.1038/srep18482

**Published:** 2015-12-21

**Authors:** Yang Liu, Siarhei Vishniakou, Jinkyoung Yoo, Shadi A. Dayeh

**Affiliations:** 1 Center for Integrated Nanotechnologies, Sandia National Laboratories, Albuquerque, New Mexico 87185, USA; 2 Department of Materials Science and Engineering, North Carolina State University, Raleigh, North Carolina 27695, USA; 3 Department of Electrical and Computer Engineering, University of California San Diego, La Jolla, California 92093, USA; 4 Center for Integrated Nanotechnologies, Los Alamos National Laboratory, Los Alamos, New Mexico 87545, USA; 5 Materials Science Program, University of California San Diego, La Jolla, California 92093, USA

## Abstract

Safe and efficient operation of lithium ion batteries requires precisely directed flow of lithium ions and electrons to control the first directional volume changes in anode and cathode materials. Understanding and controlling the lithium ion transport in battery electrodes becomes crucial to the design of high performance and durable batteries. Recent work revealed that the chemical potential barriers encountered at the surfaces of heteromaterials play an important role in directing lithium ion transport at nanoscale. Here, we utilize *in situ* transmission electron microscopy to demonstrate that we can switch lithiation pathways from radial to axial to grain-by-grain lithiation through the systematic creation of heteromaterial combinations in the Si-Ge nanowire system. Our systematic studies show that engineered materials at nanoscale can overcome the intrinsic orientation-dependent lithiation, and open new pathways to aid in the development of compact, safe, and efficient batteries.

Understanding the transport of lithium (Li) ions and electrons in the electrodes/electrolyte is crucial for achieving superior performance of lithium ion batteries (LIBs) with high energy/power density and good cyclability. These battery characteristics are highly desirable for next generation portable electronics and plug-in electric vehicles^1^,^2^. Interfaces inside the batteries, such as within the electrode materials and between electrode and electrolyte, have a critical effect on the Li ion transport^3^,^4^,^5^,^6^,^7^,^8^,^9^. Recently, surface coating on the active materials has been demonstrated to be an efficient method to improve battery performance, which essentially introduce or modify interfaces in the electrodes^10^,^11^,^12^,^13^,^14^,^15^. Grain boundaries in some polycrystalline electrodes, which can be considered as interfaces within homogeneous materials with different orientations, are found to act as facile diffusion paths for Li ions^16^,^17^.

Although interfaces can play an important role in the performance of LIBs, only few studies have explored such effects in the context of Li ion transport. Santhanagopalan *et al.* concluded that the interfaces between the electrode/electrolyte are the key limiting factor in solid-state batteries^18^. Chou and Hwang found that the interfacial Li ions at the Si/graphene interface have substantially higher mobility compared to that in bulk Si, which contributed to a higher charging/discharging rate capability^19^.

Nanoscale size effects in ionic systems, commonly known as nanoionics, are significant in ion transport, and could produce different lithiation behavior in nanomaterials compared to their bulk counterparts^20^,^21^,^22^,^23^,^24^. These behaviors that arise at nanoscale sizes and surfaces and interfaces provide a new degree of freedom for controlling the Li diffusion pathways in the LIBs and modify their lithiation behavior from intrinsic orientation-dependent lithiation^25^,^26^,^27^ to interface-dependent lithiation.

Li ion diffusion pathways can be modified to control the volume expansion direction. Large volume changes during Li insertion and extraction can generate significant strain/stress, and if not accounted for, can lead to shorting of the battery electrodes. The proper control of volume expansion direction of battery electrodes, especially for compact, light-weight, and high capacity electrodes such as Si and Ge, is an important step to mitigate the mechanical failure of the electrodes. In addition, nanostructures, such as nanoparticles, nanotubes and nanowires, are introduced with the purpose of accommodating the huge volume expansion due to the porous property or free surfaces of nanostructures^28^,^29^. The large volume expansion upon lithiation can reduce the gaps among the nanostructures and result in contact and merging/welding of materials^30^, which will change the charge transport and mechanical properties. More importantly, the Li ion diffusion path in the first lithiation stage is particularly critical, since it determines the volume expansion direction, which remains permanent during the subsequent cycles for the lifetime of the battery because of the almost isotropic lithiation/delithiation of amorphous materials generated after the first cycle. Proper control of the volume expansion direction can relieve stress accumulation, crack formation and electrode welding. Recently, we demonstrated for the first time that the Li ion transport/insertion paths at nanoscale can be tailored by interface and bandgap engineering validated via *in situ* transmission electron microscopy (TEM) electrochemistry observations^31^. These observations are key to capitalize on for the rational design of a new generation of battery electrodes.

Our work focuses on the Si-Ge materials systems which has high prospects in the development of efficient Li ion battery anodes. Si has been extensively studied as a leading candidate of anode materials for LIBs, attributed to its highest theoretical capacity, 3579 mAhg^−1^ for Li_15_Si_4_ at room temperature, which is about ten times larger than that of the commonly used graphite anode^28^,^29^,^32^. Ge, another group-IV element, has attracted additional attention in recent years, due to its much higher intrinsic electronic conductivity and about two orders of magnitude larger Li ion diffusivity compared to Si^33^. It is anticipated that the integration of Si and Ge into Si-Ge heterogeneous nanostructures can deliver a balanced and enhanced performance with optimal energy and power densities, which can be attributed to the combination of the high capacity of Si and excellent rate capability of Ge^34^,^35^,^36^. However, the kinetics of Li ion transport in Si-Ge heterogeneous nanostructures and the effects of chemical potentials that arise at the Si/Ge surfaces and interfaces on the Li ion transport are yet to be studied.

In this work, we systematically investigate the Li ion transport in various Si-Ge heterostructured nanowires (i.e. Ge/Si core/shell, Si/Ge core/shell and Si/Ge/Si core/multi-shell nanowires) using *in situ* TEM electrochemistry technique, which allows observation of the dynamic lithiation process of the nanostructured electrodes. The description of *in situ* TEM electrochemistry setup can be found in the Experimental Section. This open cell configuration provides a clean system to investigate the ionic transport at the well-defined interfaces. Our results reveal that the lithiation behavior (i.e. Li ion transport) at nanoscale can be dramatically influenced by the surface chemical potential barrier that is determined by energy band-edge line-up of the battery electrodes, which sheds light on the design of battery electrodes and architectures in the next generation LIBs.

## Results and Discussions

The different lithiation behaviors in the Ge-Si heteromaterial system can be owed to the different chemical potential barriers for Li to diffuse into either Ge or Si. The principle formulation, developed by Tersoff 37 for impurity diffusion across heterointerfaces, is that the effective chemical potential for a positively charged Li ion (Li^+^) into a semiconductor system or heterointerface can be modeled by moving a charge neutral impurity across the semiconductor surface or interface and ionizing the impurity and returning the ‘electron’ to the starting position. The chemical potential barrier for Li ion diffusion will then depend on the enthalpy of formation of Li in the material, its ionization energy, and the difference between the conduction band-edge and the electrochemical potential of the entire system. Since Li is a shallow donor in Ge and Si^38^, there will be minimal differences in ionization energies and in the enthalpy of formation in either crystal. As a result, the effective chemical potential for Li ion diffusion, 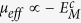, where 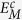 is the conduction band-edge profile in material *M*. To account for the potential barriers at the Li-Ge or Li-Si interface, we utilize known values of electron Schottky barrier heights with Si (qφ_Bn_ = −1.67 eV) and with Ge (qφ_Bn_ = −0.8 eV)^39^.

**Figure 1:**
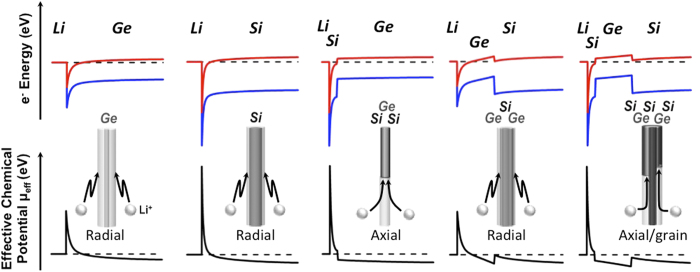
Simulated energy band-edge profiles (top row) and correspondent chemical potential barrier (bottom row) for an elemental (left two columns) and a variety of Si-Ge radial heterostructures (right three columns). The displayed chemical potential barriers dictate the Li ion diffusion pathways that were observed in experiment. Solid red is the conduction band-edge and solid blue is the valence band-edge profile. Dashed black is the equilibrium electrochemical potential.

A variety of Si-Ge heterostructured nanowires were considered in this work. Figure 1 overviews the lithiation behaviors of different Si-Ge heterostructured nanowires according to the above formulation by utilizing the correspondent simulated energy band-edge diagrams using Silvaco Atlas and the resulting chemical potential barrier for Li diffusion into these structures. For pure Si and pure Ge nanowires, Li ions can diffuse from the surface inwards and exhibit a radial lithiation behavior, resulting in unlithiated crystalline core with dark contrast wrapped by lithiated amorphous shell with a lighter gray contrast (i.e. core-shell structure). This is experimentally demonstrated in Fig. S1 in Supporting Information as well as by others and our earlier work in previous publications^40^,^41^,^42^. Although the lithiation behaviors for Si and Ge nanowires are the same (radial lithiation, core-shell structure), the Li ion experiences higher chemical potential barrier on Si surface than on Ge surface. This, together with the higher diffusivity of Li in Ge than in Si^43^, can contribute to the ease of lithiation of pure undoped Ge nanowires compared to pure undoped Si nanowires, as manifested by the much faster lithiation rate on Ge (radial lithiation rate is about 3.7 nm/s) compared to intrinsic Si (average radial lithiation rate is estimated to be less than 0.015 nm/s)^41^.

**Figure 2:**
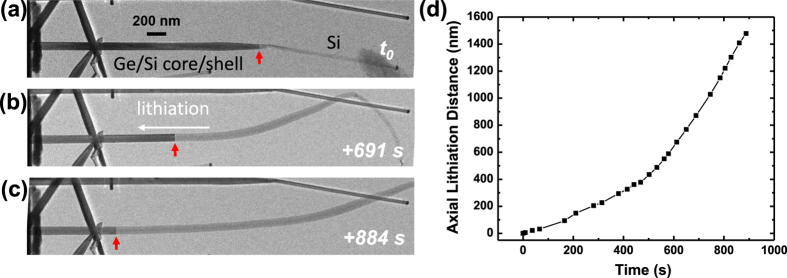
*In situ* TEM observation of Ge/Si core-shell nanowire lithiation where *t*_*0*_ in (**a**) marks the reference time at the onset of the axial lithiation with the red arrow pointing at the lithiated/unlithated or amorphous crystalline interface (ACI). (**b**,**c**) TEM images showing advancement of the ACI interface and axial elongation of the core/shell nanowire. (**d**) Plot of the axial lithiation distance as a function of time obtained from several video frames on the lithiated nanowire presented in (**a–c**).

However, when the pure Ge nanowire is coated with a conformal, epitaxial, and ultrathin Si shell (1~5 nm), lithiation behavior is dramatically changed from the radial lithiation to pure axial lithiation. Figure 2a–c and movie S1 in Supporting Information show the axial lithiation process of an individual Ge/Si core/shell nanowire (about 1 nm epitaxial Si). The reaction front is switched from a tapered shape (Fig. S1a) to lay exactly on the cross-section of the nanowire. This lithiated nanowire shows only elongation, which is in sharp contrast to the radial lithiation behavior in pure Ge and pure Si nanowires. This axial lithiation mode occurs at the beginning of lithiation (Fig. 2a). The simulated energy band-edge profile of the Ge/Si core/shell structure shown in Fig. 1 indicates that the Si coating on Ge creates an effective chemical potential barrier that is larger than that for pure Ge. As a result, lithiation occurs in a layer-by-layer fashion across the Ge core rather than through the surface across the Si shell, and this behavior was observed experimentally in our previous work^31^. The Si shell lithiation occurs after the Ge core has been lithiated. Further increase of Si shell thickness to larger than 5 nm would introduce surface roughness and grain boundary/twin formation in the Si shell, which can degrade the quality of Ge/Si interface and the crystallinity of Si shell. However, it is very interesting to assume a condition that the Si shell gets very thick and Ge core gets very thin at the same time, where a radial lithiation mode could be observed. In this case, the lithiation of Ge core may become difficult due to multiple factors including (1) increased flux of Li ion on the surface Si shell as opposed to limited Li ion flux on the Ge core, (2) lower Ge core conductivity in a thin body as opposed to higher conductivity in the Si shell, and (3) the mechanical confinement of the thick Si shell and induced compressive stresses on the Ge core which lowers the Li ion diffusivity in Ge. The interplay between these factors could determine a critical Si/Ge thickness ratio that the lithiation is switched from axial to radial lithiation.

The axial lithiation distance (i.e. reaction front migration distance) as a function of time is plotted in Fig. 2d. If the lithiation is long-range diffusion controlled, the reaction front migration distance L would be proportional to the square root of reaction time t (i.e. L ~ t^1/2^)^3^,^44^. If the lithiation is short-range interface controlled, it would give a linear relationship L ~ t^41^,^45^. The parabolic-like curve shown in Fig. 2d indicates that the lithiation speed increases as the reaction progresses, suggesting that the lithiation is impedance limited^41^. The internal resistance drops as lithiation progresses, indicating that the unlithiated [111] Ge segment close to the top kinked [112] Si segment (Fig. 2a, early stages of lithiation) has a higher resistance than that is mostly lithiated and close to the bulk Ge substrate (Fig. 2c, later stages of lithiation) where the negative potential to trigger lithiation is applied. The average layer-by-layer axial lithiation rate between 612 s to 888 s is estimated to be ~3 nm/s, which is less than the lithiation rate of pure intrinsic Ge that occurs through simultaneous lithiation of multi-layers across the surface, but is still much larger than the lithiation of pure intrinsic Si without any coating.

The impact of the effective chemical potential barrier at nanowire surfaces can be used to tailor the mechanism and rate by which Li ions diffuse into the nanowire. For the case of a Si nanowire core, we establish first the lithiation directions and rates in a Si/Ge core/shell nanowire and then demonstrate in a Si/Ge/Si core/multi-shell structure a distinct lithiation behavior from the commonly observed radial lithiation in the Ge-Si material system (in addition to the axial lithiation observed here in the Ge/Si core/shell case).

**Figure 3:**
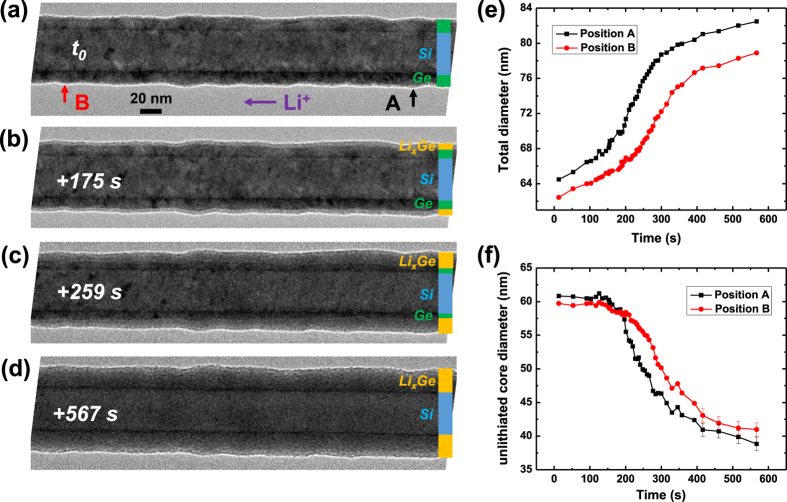
*In situ* TEM observation for lithiation of a Si/Ge core-shell nanowire shown in (**a**) where *t*_*0*_ in (**a**) marks the reference time at the onset of the core/shell lithiation and (**b–d**) show morphological evolution during the nanowire lithiation at the marked time stamps. (**e**) Plot of the measured total diameter at points A and B marked in (**a**) as a function of time showing total volume expansion. (**f**) Plot of the unlithiated core diameter for the same time period as that in (**e**).The radial lithiation rate first increases and then decreases as the reaction front/phase boundary moves closer to Si core.

Figure 3a shows the TEM image of a pristine nanowire with a Si core and a uniform 12 nm thick Ge shell (which is near the threshold thickness at which we observed conformal Ge shell coating on Si nanowires)^46^. The morphological evolution of this nanowire in the lithiation process is shown in Fig. 3b–d and movie S2 in Supporting Information. Upon lithiation, the Li ions first diffuse along the surface and react with the Ge shell, forming an amorphous Li_x_Ge layer at the outer surface of the Si/Ge core/shell nanowire. In contrast to the axial lithiation of Ge/Si core/shell nanowire, this Si/Ge core/shell nanowire displays radial lithiation, where the Li ions can enter the nanowire from the surface. This phenomenon is consistent with the lower effective surface chemical potential for Li ion diffusion into Ge compared to that into Si. Once the reaction front propagates to the surface of Si core, we observed that the lithiation stops (Fig. 3d). The total nanowire diameter change and the unlithiated core diameter change as a function of time are plotted in Fig. 3e,f, respectively. As expected, the total diameter of the nanowire monotonically increases during lithiation and the diameter of the unlithiated core monotonically decreases. The slopes of the curves first increase and then decrease, indicating that the lithiation rate increases from 0.04 nm/s (100 to 200 s) to 0.08 nm/s (200 to 400 s), and decreases again to very small value 0.02 nm/s (400 s to 550 s). The lithiation rate finally reaches almost zero as the lithiation stops at the Si surface at about 550 s. The lithiation process is initiated through the application of negative potential at the Si substrate with respect to the Li metal on the other end of the nanowire and this potential difference drives the lithiation reaction at the surface of the Si/Ge core/shell nanowire. Initially, the lithiation reaction is limited by the axial resistance of the nanowire which leads to a parabolic like increase in the lithiated length as a function of time, similar to the case of axial lithiation of the Ge/Si core/shell nanowire in Fig. 2d. As the Ge shell becomes mostly lithiated, the lithiation reaction is limited by the radial resistance which slows down the reaction rate as observed in Fig. 3e. Upon full lithiation of the Ge shell across the entirety of the nanowire length, it is anticipated that larger current will flow across the Li_x_Ge shell compared to the current flow across the amorphous crystalline interface (ACI), i.e. the Li_x_Ge/Si interface. As a result, the driving force for Li ions to diffuse into the Si nanowire core drops significantly and the lithiation halts at the surface of Si.

The above results demonstrated that the Ge/Si core/shell system is dominated by axial lithiation behavior whereas the Si/Ge core/shell system is dominated by surface radial lithiation and that heteromaterials have distinctive lithiation behavior from that of individual material components. Introducing multiple heteromaterial layers and interfaces can result in engineered effective chemical potential barriers for Li ion diffusion and possibly new behaviors that are unexplored in Li ion transport. Our excellent control over the growth in the Ge/Si material system^46–52^ enables us to explore such untested territories in nanoionics. To this end, we grew a Si layer on the Si/Ge core/shell nanowire to form a Si/Ge/Si core/multi-shell nanowire and investigated its lithiation behavior using the same methodology.

**Figure 4:**
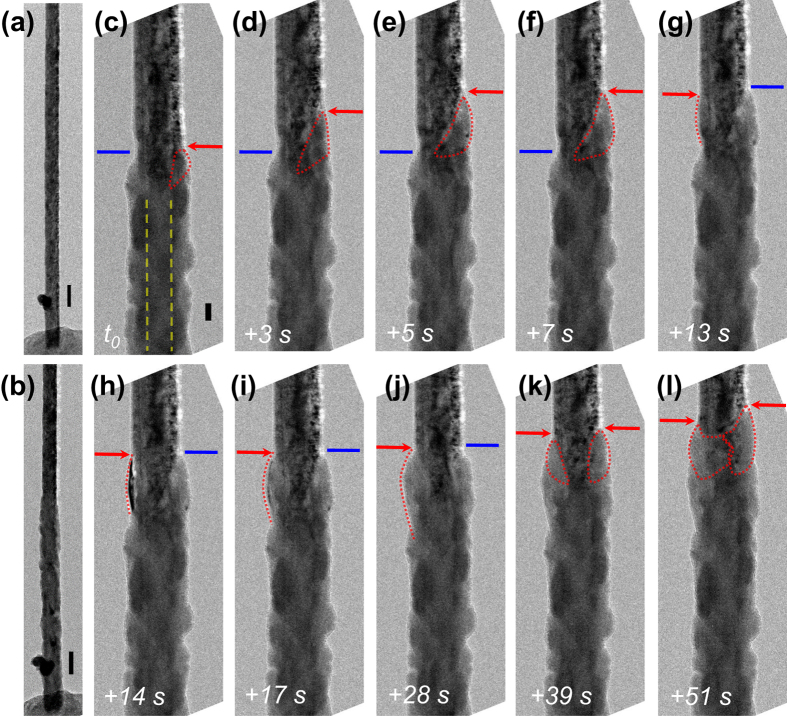
*In situ* TEM observation for lithiation of a Si/Ge/Si core/multi-shell nanowire demonstrating grain-by-grain lithiation behavior. TEM images of the nanowire before (**a**) and after (**b**) lithiation are shown in the left column. Blue line is static interface granule. Red arrow shows moving interface and expansion of lithiated granule. Yellow dashed lines show unaltered diameter of unlithiated core. Scale bar is 200 nm for (**a**,**b**) and 50 nm for all other panels.

Figure 4 shows the lithiation process of an individual 80 nm/20 nm/5 nm Si/Ge/Si core/multi-shell nanowire. In striking contrast to the pure axial elongation of Ge/Si core/shell nanowire (Fig. 2) and the radial lithiation of Si/Ge core/shell nanowire (Fig. 3), this Si/Ge/Si core/multi-shell nanowire shows a step-by-step grain-like lithiation process, as shown in Fig. 4b. The Ge shell sandwiched between the Si shell and Si core starts to lithiate first, followed by the lithiation of the outermost Si shell, as can be clearly deduced from supporting movie S3. In this case, no elongation is observed and the nanowire surface becomes relatively rough compared to the radial lithiation of Si/Ge core/shell nanowire. Figure 4c to l show magnified images with details of the lithiation process. The blue line and red arrow indicate the reaction front. Particularly, the blue line is static interface granule, and the red arrow shows moving interface and expansion of lithiated granule.

**Figure 5:**
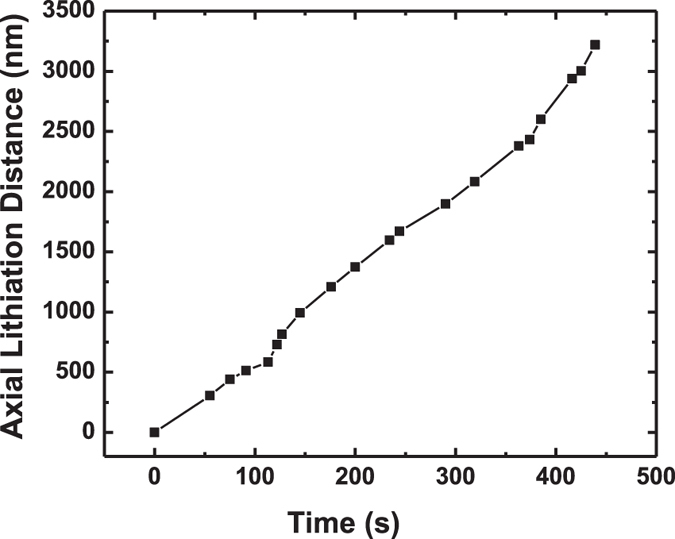
Plot of the axial lithiation distance as a function of time for the Si/Ge/Si core/multi-shell nanowire discussed of Fig. 4.

The inner Ge layer lithiation and the subsequent lithiation of the cracked Si shell is similar to that we observed in Ge/Si core/shell nanowires. The Si core in the core/multi-shell nanowire also does not lithiate, as marked by the yellow dashed line in Fig. 4c. This behavior is similar to the Si/Ge core/shell nanowire which was discussed above. The red dotted line in Fig. 4c circles a Ge grain that is starting to lithiate. Figure 4c to f exhibit the expansion of this Ge grain during lithiation. The reaction front on the right side (red arrow) of the nanowire propagates upon lithiation of this grain, while the reaction front on the left side (blue line) of the nanowire does not shift. After the grain on the right side is fully lithiated, a grain on the left side starts to lithiate, as shown in Fig. 4g–j. It is also possible that two grains on both sides are lithiated simultaneously, as exhibited in Fig. 4k,l. Since the stress generated from volume expansion of Ge upon lithiation cannot break the thick Si core axially to segment by segment, the Ge grain can only expand radially at weakest position, which cracks the Si shells that subsequently lithiate and which results in an overall rough surface. This grain-by-grain lithiation process also supports our picture that effective chemical potentials in heteromaterials influence the Li ion transport. The axial lithiation distance (measured from the closest reaction front) as a function of lithiation time is plotted in Fig. 5 and shows an almost linear lithiation behavior with an average lithiation rate of 7.1 nm/s. This grain-by-grain lithiation which facilitates Li ion diffusion along grain boundaries, is expected to be faster than the layer-by-layer axial lithiation of Ge/Si core/shell nanowires and appear to possess a unique linear relationship–indicative of kinetically limited interface reactions–in contrast to that of Ge/Si core/shell heteromaterials.

The *in situ* TEM experimental results and the qualitative analysis using energy band-edges simulations on various Ge-Si heterostructured nanowires demonstrate that interfacial chemical potential barriers play an important role in the Li ion transport and provide a simple intuitive approach to understand nanoionics in heterostructured materials, heteronanoionics. While our qualitative model suffices to interpret our systematic experimental observations, it is worth noting that it doesn’t account for the influence of strain/stress on energy band-edges and chemical potentials^53^,^54^. The nanowire lithiation is a dynamic process, where strain/stress will change with time. A more detailed model should consider the effects of strain/stress in the system during lithiation, which may provide a quantitative understanding of Li ion transport in the heterostructured nanowires. The lithiation rates can also be influenced by the choice of material and its doping level, which effectively determine the access resistance away from the lithiation front, as seen in all homo- or heteromaterial combinations we experimented in this work except for the case of grain-by-grain lithiation, where grain-boundary conduction dominates. The absolute values of the estimated lithiation rates can vary from nanowire to nanowire, due to the difficulty in controlling the impedance at the lithium oxide covering the nanowire. However, the trends of the reaction front migration (i.e. the shape of the plotted graphs) should not be greatly influenced, because they are determined by the intrinsic transport properties of the lithiated portion of the electrode materials.

Engineering materials at nanoscale encompasses multiple advantages for battery applications: (1) It provides shorter ionic transport path and higher reaction rate. The reaction characteristic time scales with square diffusion length (assuming the diffusivity is fixed in a specific material). The small size of nanomaterials (tens to hundreds of nanometers) provide shorter distances for Li ion diffusion and therefore faster lithiation rates; (2) It can accommodate volume changes and strain accompanying with lithium insertion/extraction, and thus mitigate the mechanical degradation of planar or bulk like electrodes; (3) It provides larger surface contact area with the electrolyte, thus allows a higher Li ion flux for achieving higher rate capability; (4) If the nanostructures (such as nanowires in this work) are directly grown on conductive substrate, the electrical contact between the nanostructures and current collector is greatly improved. Our work on engineering the heteromaterials (Ge/Si) extends this suite of benefits at the nanoscale for lithium ion battery electrodes and provides another degree of freedom to control the ionic transport and thus the lithiation behavior in battery electrodes, and can be adopted to other combinations of electrode heteromaterials.

## Conclusions

We investigated the Li ion transport in a variety of Ge-Si heterostructured nanowires, such as Ge/Si, Si/Ge core/shell nanowires, and Si/Ge/Si core/multi-shell nanowires, by *in situ* TEM electrochemistry. We observed pure axial lithiation in Ge/Si core/shell nanowires, radial lithiation in Si/Ge core/shell nanowires, and grain-by-grain lithiation in Si/Ge/Si core/multi-shell nanowires, which demonstrated that the effective chemical potential for Li ion diffusion can control the Li ion transport pathways in heteromaterials as supported by energy band-edge simulations and inferred chemical potential. Our findings reveal that the Li ion transport can be controlled by tailoring the material combinations in battery electrodes, which provide an additional degree of freedom in battery design for achieving superior performance and longevity. The *in situ* TEM electrochemistry experiment also establishes additional grounds to study the ionic transport in other battery systems, such as sodium (Na) ion transport in Na-ion batteries and ionic transport in multivalent batteries.

## Methods

### Materials synthesis

Ge and Si nanowires were grown on Ge (111) and Si (111) surfaces, respectively, in a cold wall CVD system (Atomate Inc.). 40 nm diameter Au colloids were used to seed the nanowire growth for all samples. The growths were carried out according to the preparation and two-step growth procedures described elsewhere^46^,^47^,^48^,^49^,^50^,^51^. Briefly stated, pure Ge nanowire elongation proceeded at ~276 °C in GeH_4_ (30% in H_2_) and GeH_4_ partial pressure, P_i-GeH4_ = 0.6 Torr and for Ge/Si core/shell nanowires with extended Si segment atop, the temperature was ramped from 276 °C to ~400 °C in GeH_4_ flow at P_i-GeH4_ = 0.15 Torr. The growth of the 1 nm Si shell and axial Si segment was carried out at ~440 °C for 1.5 min at P_i-SiH4_ = 1.4 Torr. For the Si/Ge and Si/Ge/Si core/shell NWs, the Si cores were grown at 460 ^o^C using 450 sccm SiH_4_ (50% diluted in H_2_) at 0.5 Torr pressure and the Ge shell was deposited at 410 ^o^C with a corresponding GeH_4_ partial pressure and chamber pressure are 5 mTorr and 100 mTorr, respectively. Further Si shell growth was preformed at the conditions discussed above as in the Ge/Si core/shell case.

### *In situ* TEM electrochemical lithiation setup

The *in situ* TEM electrochemical lithiation of various Ge-Si heterostructured nanowires were carried out on a Nanofactory TEM-STM piezo holder inside a FEI Tecnai F30 microscope, using an open cell configuration^55^,^56^. To fabricate a working nanobattery, a small piece of wafer with nanowires was cleaved from a large wafer substrate and was glued onto an aluminum rod using conductive epoxy to ensure good electrical contact between the nanowires and the current collector. Li metal was loaded to a tungsten rod from a freshly cut bulk Li in a helium-filled glove box (H_2_O and O_2_ concentration below 1 ppm). Both the nanowires working electrode and Li metal counter electrode were loaded on the TEM-STM holder in the same glove box. This holder was quickly transferred into the TEM column with an exposure time of about 2–5 s in air. The naturally grown Li_2_O surface on Li metal served as a solid-state electrolyte for Li ion transport. By manipulating the piezo probe inside the TEM, the Li_2_O/Li electrode was driven to contact an individual Ge-Si heterostructured nanowire to build a nanobattery. Lithiation was initiated by applying a typical potential of −2 V on nanowire electrode against the Li counter electrode.

### Energy band-edge simulations

The Silvaco ATLAS TCAD software was used to simulate the thermal equilibrium energy band structures of the heterostructured nanowires discussed in this work. For all cases under consideration, the nanowire core was fixed at 60 nm and undoped material combinations have been used.

## Additional Information

**How to cite this article**: Liu, Y. *et al.* Engineering Heteromaterials to Control Lithium Ion Transport Pathways. *Sci. Rep.* **5**, 18482; doi: 10.1038/srep18482 (2015).

## Supplementary Material

Supplementary Information

Supplementary Movie S1

Supplementary Movie S2

Supplementary Movie S3
