# Supplementary material for: Engineering Heteromaterials to Control Lithium Ion Transport Pathways
Source: Sci Rep. 2015 Dec 21;5:18482. doi: 10.1038/srep18482 (PMC4685276; doi:10.1038/srep18482)
Supplement: Supplementary Information [file srep18482-s1.pdf]

Supplementary Information

Engineering Heteromaterials to Control Lithium Ion Transport Pathways

Yang Liu[1,2], Siarhei Vishniakou[3], Jinkyoung Yoo[4], and Shadi A. Dayeh[3,5*]*

[1]Center for Integrated Nanotechnologies, Sandia National Laboratories, Albuquerque, New Mexico 87185, USA

[2]Department of Materials Science and Engineering, North Carolina State University, Raleigh, North Carolina 27695, USA

[3]Department of Electrical and Computer Engineering, University of California San Diego, La Jolla, California 92093, USA

[4]Center for Integrated Nanotechnologies, Los Alamos National Laboratory, Los Alamos, New Mexico 87545, USA

[5]Materials Science Program, University of California San Diego, La Jolla, California 92093, USA

[*]Corresponding authors: yliu78@ncsu.edu (Y.L.), sdayeh@ece.ucsd.edu (S.A.D.)

Supporting Figures

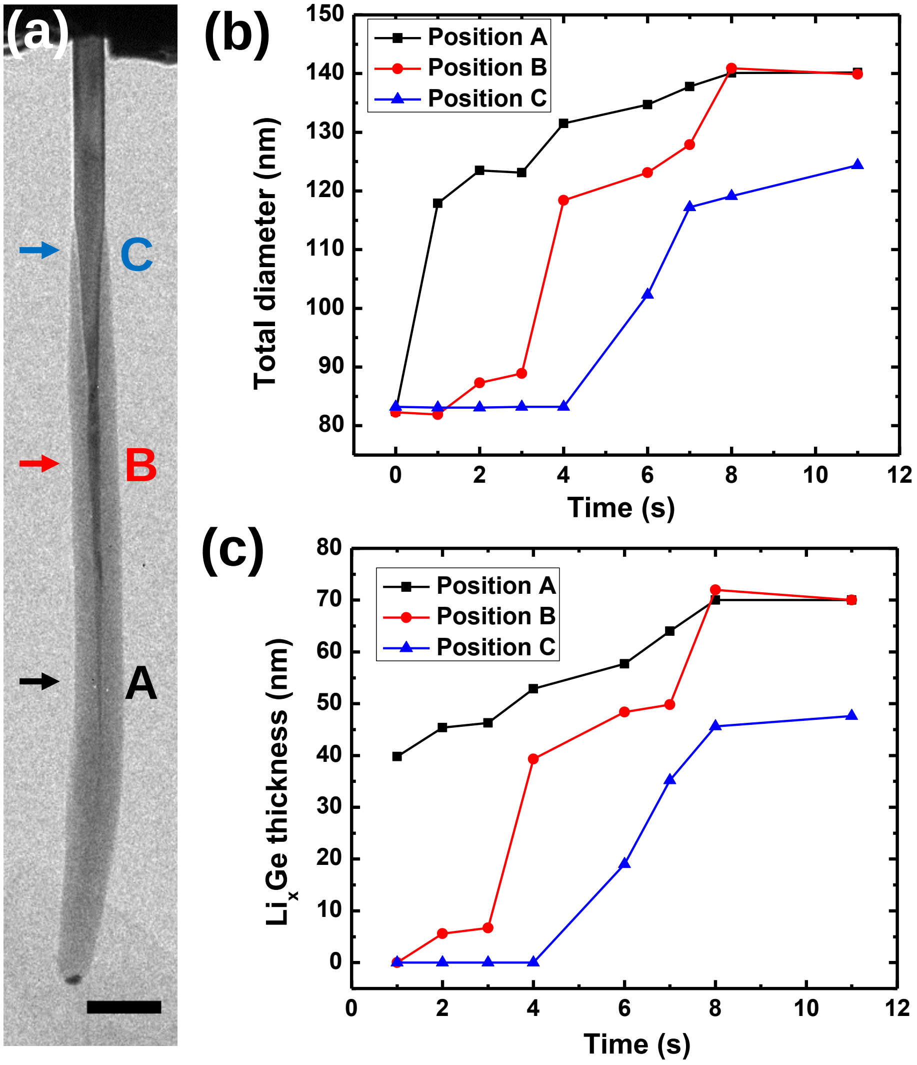

Figure S1. Core/shell lithiation of a pure Ge nanowire and the lithiation kinetics. (a) In-situ TEM image showing the core/shell lithiation behavior of a pure Ge. Plot of the total diameter (b) and Li_xGe thickness (c) of the pure Ge nanowire shown in (a) as a function of time, showing the very fast radial lithiation kinetics. Scale bar: 100 nm.

Supporting Movies

Movie S1: Lithiation of a Ge/Si core/shell nanowire, showing the axial lithiation behavior. The movie was recorded at 2 frames/second and is played at 40x speed.

Movie S2: Lithiation of a Si/Ge core/shell nanowire, showing the core/shell lithiation behavior. The movie was recorded at 3 frames/second and is played at 40x speed.

Movie S3: Lithiation of a Si/Ge/Si core/multi-shell nanowire, showing the grain-by-grain lithiation behavior. The movie was recorded at 3 frames/second and is played at 15x speed.
